# Individual Effect of Bull Prevails over Sperm Characteristics in Predictive Models

**DOI:** 10.3390/biom16040581

**Published:** 2026-04-14

**Authors:** Adriano Felipe Perez Siqueira, Leticia Signori de Castro, Thais Rose dos Santos Hamilton, Vivian Cardoso Castiglioni, Luana de Cássia Bicudo, Tamie Guibu Almeida, Rodolfo Daniel Mingoti, Camilla Mota Mendes, Roberta Leite, João Diego de Agostini Losano, Marcilio Nichi, Mayra Elena Ortiz D’Avila Assumpção

**Affiliations:** 1 Laboratory of Sperm Biology, Department of Animal Reproduction, School of Veterinary Medicine and Animal Science, University of São Paulo, São Paulo 05508-270, SP, Brazil; adrianofps@gmail.com (A.F.P.S.); castro.lsc@gmail.com (L.S.d.C.); thais.hamilton@unesp.br (T.R.d.S.H.); vivianscp@gmail.com (V.C.C.); luanacbicudo@gmail.com (L.d.C.B.); tamieguibu@gmail.com (T.G.A.); rodolfo.mingoti@gmail.com (R.D.M.); camillam@usp.br (C.M.M.); 2 Laboratory of Embryology, Department of Animal Morphology and Physiology, School of Agriculture and Veterinary Sciences, São Paulo State University, Jaboticabal 14884-900, SP, Brazil; 3 Laboratory of Andrology, Department of Animal Reproduction, School of Veterinary Medicine and Animal Science, University of São Paulo, São Paulo 05508-270, SP, Brazil; bobbieleite@gmail.com (R.L.); jdalosano@gmail.com (J.D.d.A.L.); mnichi@usp.br (M.N.)

**Keywords:** in vitro fertility, CASA, flow cytometer

## Abstract

Sperm quality influences bovine in vitro embryo production (IVEP). Linear regression is a statistical tool that models the relationship between a dependent variable and one or more independent variables. It can be used to predict outcomes, analyze trends, and understand the impact of variables. These models are useful for indicating which sperm variables most influence IVEP results, facilitating the selection of superior samples to enhance IVEP. Using early IVEP indicators, such as cleavage rate, can assist in scheduling recipient preparation. This work aimed to construct linear regression models to study the influence of a comprehensive set of sperm variables and cleavage rate on IVEP yields. A dataset comprising 51 semen batches from 23 Nellore bulls was compiled, including 26 sperm variables from computer-assisted sperm analysis (CASA) and flow cytometry per batch, with 184 IVEP procedures. The most robust predictive model had a coefficient of determination of 0.6358; furthermore, the BULL variable was the most influential predictor, yielding an independent coefficient of determination of 0.5218. Models that were exclusively founded on sperm analysis yielded meager coefficients of determination (<0.04). However, to predict the best batch from a bull, individual models achieve coefficients of determination ranging from 0.58 to 0.99. Contributions, impacts, and positive or negative correlations of various sperm variables with in vitro performance were influenced by the bull. We conclude that the BULL variable was the dominant predictor of in vitro performance, with cleavage rates serving as an early estimator of blastocyst rates. The predictive utility of analyzed sperm traits remains limited. Nonetheless, individualized models offer a valuable tool for selecting optimal batches for preferred bulls within IVEP laboratories, culminating in heightened blastocyst rates.

## 1. Introduction

Bovine in vitro embryo production (IVEP) is a valuable reproductive technique that facilitates the utilization of oocytes from genetically superior cows and young heifers that might otherwise be unused [1]. This approach accelerates genetic advancements and diminishes generation intervals [2]. Nevertheless, oocytes subjected to IVEP are less likely to reach the blastocyst stage than their counterparts under in vivo conditions [1,2,3]. However, combining IVEP with oocyte retrieval through ovum pick-up yields, in Nellore cattle, more oocytes, leading to more embryos and pregnancies than in vivo sources per time unit [4]. These distinct advantages have positioned IVEP as the primary source of embryos in several nations [5].

This technique allows multiple embryos to be generated from a single semen straw, making it crucial for scenarios involving valuable semen, such as those sourced from high genetic merit bulls or sex-sorted samples, to optimize costs per embryo [6]. Semen samples for in vitro fertilization (IVF) are pivotal in the variability observed in IVEP success rates. Disparities in in vitro performance among bulls and even among batches from the same bull are well documented [7,8,9,10,11,12,13,14,15,16]. For example, Palma and Sinowatz’s (2004) [13] reported that blastocyst rates varied from 6.9 to 51.2% depending on the bull selected for IVF. Similarly, Castro et al. (2018) [17], in 5000 IVEP procedures involving 500 bulls, identified various blastocyst rates based on distinct bulls, ranging from >30% (high-performance bulls) to <19% (low-performance bulls). These findings emphasized the potential impact of selecting bulls with superior in vitro performance, thereby enhancing the efficiency of IVEP.

However, conducting IVEP solely to assess the in vitro performance of a semen sample would be costly and labor-intensive. It would be considerably advantageous if in vitro performance could be predicted through analysis of key sperm functions and/or cellular structures. This approach aligns logically with the essential roles of these attributes during fertilization and embryo development [18]; in that regard, motility [10,14,15,16,17,18], acrosome and membrane integrity [8,9,19,20], mitochondrial status [10,18], and chromatin integrity [17,21] are key attributes associated with cleavage rate and blastocyst production.

Numerous studies have tried to relate sperm traits to in vitro performance and their potential as predictive indicators across various species, including cattle [22,23,24,25], pigs [26,27], sheep [28], and humans [29,30]. However, the connection between sperm traits and in vitro performance has yielded inconsistent outcomes [22,23,24,25,30,31,32]. In the latter scenario, confounding effects could arise, e.g., reliance on a single bull potentially hindering accurate assessment of individual bull effects.

Effective fertilization and subsequent embryo development necessitate the proper functioning of several sperm traits. Consequently, employing a combination of methods could offer a viable alternative for accurate prediction of fertilization potential of semen samples [33]. A model integrating multiple traits via both flow cytometry analysis and computer-assisted sperm analysis (CASA) was outlined by Sellem and colleagues (2015) [34] for field fertility data. With this context in mind, the present study aimed to construct predictive models for IVEP, utilizing an array of sperm variables encompassing CASA and cytometry analysis. Our objective was to identify specific sperm traits that contribute and quantify their contributions toward predicting the in vitro performance of sperm samples.

## 2. Materials and Methods

Ethical Approval: This study was approved by the Ethics Committee for Animal Use at the School of Veterinary Medicine and Animal Science of the University of São Paulo (Protocol Number: 2720/2012). All experiments were performed following relevant guidelines and regulations.

Reagents and Solutions: Unless otherwise specified, all chemical reagents and solutions utilized in this study were obtained from Sigma–Aldrich (St. Louis, MO, USA).

### 2.1. Experimental Design

**Figure 1:**
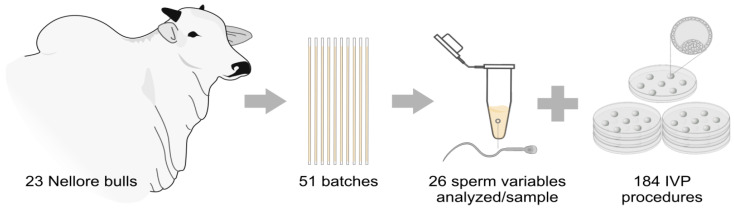
Experimental design—construction of the database.

Construction of the IVP Database I (Figure 1): Semen samples were obtained from various Insemination Centers, comprising 51 batches from 23 Nellore bulls. All samples were cryopreserved using egg-yolk extenders. Each batch underwent detailed analysis of 26 sperm traits via computer-assisted sperm analysis (CASA) and flow cytometry, performed after thawing and both before and after Percoll^®^ selection. For each semen batch, 3 to 4 in vitro embryo production (IVEP) procedures were conducted, totaling 184 IVEP assessments. Cleavage and blastocyst rates were evaluated for each procedure. A single bull with a consistently high blastocyst rate served as a quality control in every IVEP session. Only IVEP data from procedures where the control bull achieved blastocyst rates greater than 20% were included; any procedures failing this criterion were repeated.

In vitro embryo production (IVEP):

The IVEP procedures were conducted by the established protocol of our laboratory, as described by Siqueira et al. [18]. In summary, ovaries obtained from an abattoir were transported to the laboratory within 2–6 h after collection and immersed in saline solution (0.9% at 30 °C). Follicles within the 2–8 mm diameter range were aspirated via a 21-gauge needle connected to a 5 mL syringe. Aspirated follicular fluid was transferred to 15 mL conical tubes and maintained in a water bath at 37 °C for 10 min. The resulting pellet at the bottom was recovered and placed in a Petri dish. Cumulus–oocyte complexes (COCs) were meticulously identified and evaluated. COCs exhibiting intact cumulus cell layers and uniform cytoplasm were chosen. These COCs were subjected to 3 successive washes in a holding medium (TCM199 HEPES supplemented with 10% fetal calf serum (FCS, Gibco, South America), 22 μg/mL sodium pyruvate, and 50 μg/mL gentamicin). The samples were subsequently washed 3 times in maturation medium (TCM199 bicarbonate supplemented with 10% FCS, 22 μg/mL sodium pyruvate, 50 μg/mL gentamicin, 0.5 μg/mL FSH Folltropin V from Vetrepharm, Inc., Belleville, ON, Canada, and 50 μg/mL hCG from Vetecor Laboratories, Calier, Spain, along with 1 μg/mL 17β-estradiol). Oocytes were pooled from multiple ovaries collected over time and randomly allocated across bulls and procedures to minimize female-related variability. Although donor and collection day effects were not explicitly modeled due to a lack of individual data, this randomization reduces potential biases. After these washes, batches of 20 COCs were positioned in 90 μL maturation medium droplets and subsequently covered with mineral oil. This maturation process occurred for 22–24 h at 38.5 °C and 5% CO_2_ in air under high humidity.

For in vitro fertilization (IVF), COCs that had matured in vitro were subjected to 3 washes in pre-IVF medium (TCM199 HEPES supplemented with 0.003% BSA-V, 22 μg/mL sodium pyruvate, and 50 μg/mL gentamicin), followed by 3 washes in Fert-TALP medium [35]. Subsequently, batches of approximately 20–30 mature COCs were placed in 90 μL Fert-TALP droplets and covered with mineral oil. These droplets were then inseminated on Day 0 (D0) with 5 μL of prepared sperm (resulting in a final concentration of 1.25 × 10^6^ motile sperm/mL) and co-incubated for 20 h at 38.5 °C and 5% CO_2_ in the air under high humidity conditions. In each IVP procedure, 4 droplets were inseminated via sperm samples, resulting in 60–90 COCs analyzed per sample. The 51 semen samples (batches) were randomly distributed across each IVP procedure.

In vitro culture (IVC—Day 1):

Following in vitro fertilization (IVF), inseminated oocytes underwent gentle denudation through repeated pipetting in pre-IVF medium, followed by 3 subsequent washes. The presumed zygotes were subsequently subjected to three washes in KSOM medium (Millipore Corporation, New Bedford, MA, USA), organized into groups of 20–30 structures, and then placed within 60 μL KSOM droplets under a layer of mineral oil. These droplets were then cultured for 8 days and maintained at 38.5 °C in an atmosphere composed of 5% CO_2_, 5% O_2_, 90% N_2_, and high humidity.

During IVC on the third day (D3), 30 μL of KSOM medium was drawn from each droplet and substituted with 30 μL of KSOM medium supplemented with 10% (*v*/*v*) fetal calf serum (FCS), constituting 5% of the final concentration. On the fifth day, 30 μL of KSOM supplemented with 5% (*v*/*v*) FCS was added to each droplet.

The cleavage rate (number of cleaved embryos/total inseminated oocytes × 100) was evaluated on day 3 (D3). The blastocyst rate (number of blastocysts/total inseminated oocytes × 100) was subsequently documented on day 8 (D8). Embryo development rate (amount of blastocysts/number of cleaved embryos × 100) was defined as the proportion of embryos that achieved an early blastocyst stage or beyond, considering the total number of cleaved embryos.

Semen Preparation:

Commercial semen straws acquired at Insemination Centers were thawed by immersion for 30 s in a water bath set at 37 °C. Before selection, motility before selection (MB) was assessed through phase contrast microscopy by the same evaluator. A slide warmed at 37 °C was utilized, with a 5 μL drop of semen placed on it, which was subsequently covered by a heated coverslip. To select motile sperm, the contents of the straws were subjected to centrifugation on a discontinuous Percoll^®^ density gradient (400 μL Percoll^®^ 45% (*v*/*v*) layered over 400 μL Percoll^®^ 90% (*v*/*v*), prewarmed to 38.5 °C for 6 min at room temperature and 9000× *g*. Subsequently, 100 μL of the pellet resulting from this process was retrieved and then washed via centrifugation in 1 mL of Fert-TALP solution prewarmed to 38.5 °C [35]. The washing step was conducted for 3 min at room temperature at 9000× *g*, without capacitation-inducing factors. The recovered cells at the bottom of this washing process were utilized for subsequent analyses. Next, motility after selection (MA) was evaluated as described, and sperm count was determined via a Neubauer chamber to determine sperm concentration (CONC). After sperm counting, a volume of Fert-TALP solution devoid of capacitation-inducing agents was added to the samples; this volume is referred to as the added volume (AV). This addition was done to adjust the sperm concentration to a final concentration of 25 × 10^6^ motile spermatozoa/mL. Following this concentration adjustment, the total volume of the sample, which includes the original pellet volume plus the added volume, was defined as the final volume (FV). After this preparation step, the samples were divided for use in IVF insemination and semen analysis.

### 2.2. Semen Analysis

Flow cytometry was used to concurrently evaluate the integrity of the acrosome (AI) and plasma membrane (PI) in the same sample. For this assessment, 187,500 sperm were incubated for 5 min with 10 μM propidium iodide and 5 μg of fluorescein-conjugated *Pisum sativum* lectin (FITC-PSA). Propidium iodide emits red fluorescence upon encountering damaged plasma membranes, whereas FITC-PSA emits green fluorescence upon interaction with damaged or reacted acrosomes.

To gauge the mitochondrial membrane potential, another set of 187,500 sperm was incubated for 5 min with 1 μM tetraethyl benzimidazole carbocyanine iodide (JC-1) [18]. This probe elicits green fluorescence in the presence of low mitochondrial membrane potential and red/orange fluorescence in the presence of high mitochondrial membrane potential (HMMP). The index representing HMMP from these samples was calculated and subsequently incorporated into the predictive model.

Chromatin status analysis was conducted with an established protocol [17]. Briefly, 375,000 prepared cells were combined with 50 μL of TNE buffer (10 mM Tris-HCl, 0.15 M NaCl, 1 mM EDTA disodium, pH = 7.4) and 100 μL of acid detergent solution (0.08 M HCl, 0.15 M NaCl, 0.1% (*v*/*v*) Triton^TM^ X-100, pH = 1.4). After 30 s, 300 μL of acridine orange solution (6 μg/mL in 0.1 M citric acid, 0.2 M Na_2_HPO_4_, 1 mM EDTA, 0.15 M NaCl, pH = 6.0) was added, and acquisition took place 3–5 min later through flow cytometry. The index of chromatin denaturation (CD) following acid challenge was quantified and incorporated into a predictive model.

Control Experiments and Flow Cytometry Analysis:

To better analyze the results, 2 controls were applied. Control 1 considered good samples submitted only to a Percoll^®^ gradient; Control 2 considered samples with damaged acrosomes, damaged membranes, and the absence of mitochondrial membrane potential. To obtain Control 2, a straw underwent a series of freezing/thawing cycles (1 min in liquid nitrogen followed by 1 min in a water bath at 60 °C, repeated 5 times). Furthermore, to account for Control 2 for chromatin damage, a sample was subjected to a 1 min incubation with HCl (1.2 M) in an acid detergent solution at a pH of 0.1.

All flow cytometry analyses were conducted with a Guava EasyCyte Mini System (Guava^®^ Technologies, Hayward, CA, USA) equipped with a 20 mW 488 nm argon excitation laser.

FlowJo software (version 10.2 Flow Cytometry Analysis Software–Tree Star Inc., Ashland, OR, USA) was used to analyze cytometry data. Compensation parameters remained consistent across all samples. A total of 20,000 events were assessed per sample. Controls 1 and 2 (sperm without damage and sperm with induced damage, respectively) were subjected to various ratios (1:3, 1:1, 3:1) and were mixed accordingly. Controls, along with their respective proportions, were meticulously analyzed. The cells were identified and chosen for analysis, excluding debris, probe particles, and non-single-sperm events. A gate was applied to the forward scatter (FSC) vs. green fluorescence dot plot (log mode) to isolate single sperm events for acrosome, membrane, and mitochondrial analyses. In the case of chromatin analysis, the gate was applied to the forward scatter (FSC) vs. red fluorescence dot plot (log mode). Negative and positive thresholds and gates were established for each analysis to optimize the determination coefficient during control and proportion analyses. These controls and proportion analyses were performed individually using samples from three different bulls. For each of these bulls, the determination coefficients achieved for all analyzed traits exceeded 0.96 (Supplementary Material Data). The gating configurations derived from these controls were then consistently applied across all samples.

First, events acquired for the evaluation of acrosome, plasma membrane, and mitochondrial membrane potential were analyzed in a green fluorescence (log mode) versus forward scatter (log mode) dot plot. A rectangular gate was applied to select events corresponding to sperm cells. Events acquired for chromatin status assessment were analyzed using red fluorescence (log mode) versus forward scatter (log mode) dot plot. Selected events were subsequently evaluated using histograms: green fluorescence for acrosome evaluation, red fluorescence for plasma membrane integrity, and yellow fluorescence for mitochondrial membrane potential. For chromatin status evaluation, selected events were analyzed using a histogram of alpha T Y (log), a derived parameter defined as the ratio of yellow fluorescence to total fluorescence (yellow + green fluorescence) as previously described by our group [17].

Computer-assisted sperm analysis:

Sperm kinematics were objectively assessed via computer-assisted sperm analysis (CASA System, IVOS version 12.2; Hamilton Thorne, Beverly, MA, USA). Drops (5 µL) from samples were placed onto slides warmed at 37 °C and covered with corresponding coverslips. A minimum of 6 fields and 1000 cells were assessed on a 37 °C heated stage by acquiring 60 frames/s (Hz), a minimum contrast of 60, a minimum cell size of 5 pixels, a path velocity (VAP) of 50 µm/s and a straightness (STR) of 70% for progressive cells, a slow-cell VAP cutoff of 30 µm/s and a VSL cutoff of 15 µm/s. Fields of view were examined to determine the following: total motility (TM—%); progressive motility (PM—%); oscillation rate wobble (WOB), average path velocity (VAP—μm/s); straight-line velocity (VSL—μm/s); curvilinear velocity (VCL—μm/s); lateral head displacement (ALH—μm); beat cross frequency (BCF—Hz); straightness (STR—% = [VSL/VAP] × 100); and linearity (LIN—% = [VSL/VCL] × 100). For each of these parameters, average values of fields and individual values for each sperm sample were obtained for subsequent analysis of kinetic subpopulations, as described by Núñez-Martínez et al. [36]. Sperm were categorized into groups: rapid (VAP > 50 μm/s), medium (30 μm/s < VAP < 50 μm/s), slow (VAP < 30 μm/s or VSL < 15 μm/s), and static [37].

Blastomere Cell Count:

Blastocysts on day 8 of in vitro culture (IVC) were fixed in 4% paraformaldehyde (*v*/*v* in PBS) for 30 min. Samples were subsequently washed 3 times in PBS with 1% (*w*/*v*) polyvinylpyrrolidone (PVP) and stored in PBS at 4 °C. For analysis, embryos were incubated in a permeabilization solution (0.5% Triton, 1% PVP in PBS) for 2 h at room temperature. Following permeabilization, embryos were washed 3 times in PBS with 1% PVP and then incubated with Hoechst 33342 (5 μg/mL) for 10 min. Embryos were placed on slides, covered with coverslips, and subjected to epifluorescence microscopy. Images were acquired in the blue channel (Hoechst 33342; excitation = 350 nm, emission = 460 nm) for blastomere counting. Blastomere number was measured as the average per embryo per IVEP procedure to maintain statistical independence. The number of blastomeres in each embryo was used as a dependent variable within the predictive model.

### 2.3. Statistical Analyses

The Statistical Analysis System (SAS), version 9.4 for Windows, was used, via the Enterprise Guide (version 7.1) interface. The dataset was composed of 184 in vitro embryo production (IVEP) procedures originating from 51 semen batches belonging to 23 *Bos indicus* bulls. The database included 26 sperm trait variables, which included motility values (obtained through CASA), sperm kinetics subpopulations, flow cytometry measurements, and sperm preparation analysis pertinent to in vitro fertilization (IVF). These variables were categorized as shown in Table 1.

Sperm subpopulation—To discern kinetic subpopulations, a 3-step statistical process was used [36]. In the first step, a principal component analysis (PCA) was performed to summarize the variables. The VARIMAX method with Kaiser normalization was applied as the rotation method. In the second step, a non-hierarchical K-means clustering procedure was conducted to define cluster centers based on Euclidean distances. A hierarchical dendrogram was subsequently used to determine the optimal number of clusters, using variables selected from the PCA. The third step consisted of a chi-square analysis to compare subpopulations. All statistical analyses were performed using Statgraphics Centurion XVI^®^ (Statpoint Technologies Inc., Warrenton, VA, USA), and differences were considered significant at *p* < 0.05. A total of four subpopulations (SUB1, SUB2, SUB3, and SUB4) were identified based on sperm kinetic characteristics, as described in Table 2.

These variables were subjected to appropriate statistical analyses as per the research objectives.

The GLMSELECT procedure in SAS was used to construct a general linear model. This model is exploratory, identifying variables with potential and may be sample-dependent. The selection process was carried out via the STEPWISE selection method. A significance level of <0.25 was used to introduce variables into the model, whereas a significance level of <0.10 was used to retain variables within the model. Criteria for variable selection in the model were based on the value of the adjusted coefficient of determination (ADJRSQ).

## 3. Results

In vitro Embryo Production Database: Broad Spectrum of Bull Performance

**Figure 2:**
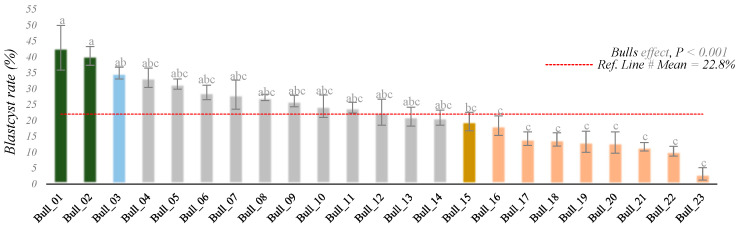
Blastocyst (% and SEM) distribution among the 23 bulls. Ref. Line #Mean = 22.8%. ^a–c^ Means without a common letter differed (*p* < 0.001).

Embryos were generated via IVEP across all 51 batches derived from the 23 selected Nellore bulls, totaling 184 procedures. Average rates of cleavage and blastocyst formation were 74.83% and 22.8%, respectively. However, the range of average blastocyst rates was large, varying from 3.2 to 42.9% among bulls (bull effect, *p* < 0.001; Figure 2).

### 3.1. Development of Predictive Models for Blastocyst Rate

The complete model had a good determination coefficient (0.6358; Table 1). Upon removing the categorical variable BULL, we created an alternative predictive model with a slightly diminished determination coefficient of 0.4321 (Table 3). Nonetheless, this second model retained the independent variable cleavage rate. In our findings, the individual effect of the animal represented by the categorical variable BULL emerged as the most potent predictor. This conclusion was supported by assessing the predictive capacity of the categorical variable BULL alone, which resulted in a determination coefficient of 0.5218 (Table 3). All predictive models with their respective determination coefficients are in Supplementary Material Data, Table S1.

### 3.2. Building Predictive Models for Embryo Development

Predictive models for embryo development (number of blastocysts/number of cleaved embryos × 100) yielded analogous results to those observed for blastocyst rates, with the variable BULL emerging as the foremost predictive factor (Table 3). The complete model exhibited a determination coefficient of 0.52, whereas the solitary inclusion of the variable BULL attained a coefficient of 0.48. Surprisingly, the cumulative influence of all the examined sperm traits produced a meager determination coefficient of merely 0.05 (Supplementary Material Data—Table S2).

### 3.3. Building Predictive Models for Cleavage Rate

Predictive models for cleavage rate yielded intriguing results, with a determination coefficient lower than that observed for the blastocyst rate prediction (Table 3). Like the patterns observed for blastocyst rates, BULL emerged as the primary predictor for cleavage rate. The complete predictive model had a determination coefficient of 0.3576, whereas the variable BULL in isolation achieved a coefficient of 0.2775, and the model without the variable BULL had a low coefficient of 0.0230. Predictive models constructed with various sperm traits and their determination coefficients are in the Supplementary Material Data (Table S3).

### 3.4. Building Predictive Models for the Number of Embryo Cells

Given the robust associations of the variable BULL and sperm traits with embryo development and blastocyst rates, we constructed a predictive model to estimate the number of embryo cells—indicative of embryo quality and developmental potential (Table 3). In a departure from our previous findings, the variable BULL did not have any discernible effect on the number of blastocyst cells. Moreover, predictive models built solely on sperm analysis yielded determination coefficients < 0.13 (Table S4).

### 3.5. Building Individual Predictive Models for the Blastocyst Rate per Bull

Given the dominant predictive power of the variable BULL and the significant decline in predictive accuracy in its absence, individual predictive models were developed for each specific bull (Table 4). This study aimed to determine whether sperm trait analysis could effectively predict in vitro performance of new batches from bulls previously subjected to IVEP. To this end, we targeted bulls with a more extensive history of different batches in our dataset (3 or 4 batches per bull). It was noteworthy that the determination coefficients of these individual models ranged from 0.59 to 0.99 and surpassed those of the general models.

## 4. Discussion

Testing 51 batches from 23 randomly selected Nellore bulls under identical in vitro embryo production (IVEP) conditions and using a shared pool of oocytes to reduce female-related variability, there was significant variation in blastocyst rates among bulls (3.2 to 42.9%). This outcome aligned with the findings of Palma and Sinowatz [13] and Castro et al. [17] and highlighted the potential to increase in vitro embryo production by up to 10-fold if a reliable tool, e.g., predictive models, were developed to select superior bulls.

To establish a robust predictive model, Utt [33] emphasized that dependent and independent variables should have a dynamic range, a criterion that our dataset fulfilled. Additionally, Utt [33] advocated for randomization of bulls rather than deliberate selection, as the latter could distort fertility models and lead to inaccurate conclusions. Previous studies assessing the predictive capability of sperm traits have typically focused on bulls categorized into high- and low-fertility groups based on in vitro performance. In contrast, our study included 51 randomized semen samples with unknown in vitro performance profiles (data not presented), creating a comprehensive dataset that captured a broad spectrum of IVEP outcomes.

A common limitation in previous investigations is that sperm analysis data were often collected from different batches than those used to determine fertility rates. This disconnection introduces substantial variability, ultimately compromising the effectiveness of predictive models [33]. To address this issue, we analyzed sperm from the same batch—and even the same straw—used for IVEP, ensuring a strong linkage between sperm measurements and fertility data. Despite our efforts to control limiting factors such as sample selection and data consistency, thereby providing a solid foundation for predicting in vitro semen performance, a useful predictive model could not be achieved in this study, as discussed below.

There were no significant differences among bulls in cleavage rates, indicating that fertilization was not the primary factor contributing to the low blastocyst rates in some bulls. These findings aligned with Ortega et al. [38], who also reported no variation in cleavage rates among bulls with differing conception rates. A predictive model for blastocyst rates incorporating cleavage as a variable yielded a determination coefficient of 0.4321, lower than that reported by Zhang et al. [39] (r = 0.81). While cleavage rate is a post-fertilization variable and thus cannot be used for pre-IVF semen selection, its inclusion in predictive models serves as an early estimator of blastocyst development, providing practical utility for IVEP laboratories. However, including cleavage rate in models intended for predicting blastocyst rates from sperm traits alone creates a temporal circularity problem, as cleavage is measured after fertilization. Therefore, predictive models excluding cleavage rate are more appropriate for pre-IVF semen evaluation, as demonstrated by our analyses. Although models incorporating cleavage data can assist IVEP laboratories in estimating blastocyst development for embryo transfer planning and recipient preparation, such models do not align with the primary objective of this study, which was to predict in vitro bull performance based solely on sperm trait analysis. Regrettably, predictive models relying solely on sperm traits as independent variables had poor predictive power. None of the individual sperm traits yielded a substantial determination coefficient; furthermore, even when all significant variables were combined, the model’s determination coefficient was <0.04.

The modeling approach adopted in this study should be interpreted within an exploratory framework. The use of stepwise selection based on adjusted R^2^ may introduce instability in variable selection and optimistic bias in coefficient estimation. In addition, the absence of internal or external validation procedures, such as cross-validation or bootstrap resampling, limits the generalizability and predictive robustness of the models. Therefore, the results should be interpreted as hypothesis-generating rather than as evidence of predictive capacity.

The dependent variables analyzed in this study, including cleavage rate, blastocyst rate, and embryo development rate, are proportion-based outcomes derived from counts of biological events. Although linear models were used to allow comparability across analyses, this approach may not fully account for the distributional properties of these variables, particularly regarding boundedness and potential heteroscedasticity. Alternative approaches, such as binomial or beta regression models, may provide a more appropriate statistical framework and should be considered in future studies.

Previous studies reported higher determination coefficients when combining sperm traits to predict field fertility. For example, Sellem et al. [34] reported a 0.40 determination coefficient using a model based on CASA and flow cytometry data. In Buffalo, Singh et al. [40] achieved a coefficient of 0.73 using lipid peroxidation, acrosome, and plasma membrane integrity variables. Gliozzi et al. [41] developed a model with a determination coefficient of 0.47 by incorporating kinetics and DNA sperm variables, whereas Kumaresan et al. [42] achieved 0.83 using membrane integrity, oxidative stress, and DNA parameters. Additionally, Narud et al. [43] reported a coefficient of 0.59 using a model that relied on a single cytometry variable and 3 metabolite analysis variables. These variations highlighted the complexity and variability inherent in predicting fertility outcomes based on sperm traits.

It is crucial to emphasize that field and in vitro fertility exhibit significant disparities. Al Naib et al. [31] reported that bulls with different in vivo fertility rates had no differences in blastocyst rates during in vitro embryo production (IVEP). Similarly, Vandaele et al. [44] reported negative correlations between bulls’ in vivo and in vitro fertility. More recently, Ortega et al. [38] confirmed that bulls with varying conception rates exhibited no differences in cleavage rates under IVEP conditions. This dissociation between in vivo and in vitro fertility has been corroborated by other studies [13,14,39,45]. These findings suggest no correlation between in vitro and in vivo fertility; in some situations, high in vivo fertility may exhibit lower in vitro fertility.

To the best of our knowledge, only 1 recent study has addressed the prediction of in vitro fertility [46]. However, that study employed a different methodology. The authors used logistic regression analysis to classify Senepol bulls into 3 in vitro fertility groups (low, medium, or high embryo production) based on sperm analysis. Remarkably, only motility subpopulations from flow cytometry and computer-assisted sperm analysis (CASA) were incorporated into the model for classification. Additionally, the study utilized artificial neural networks (ANNs) and artificial intelligence (AI), a machine learning approach capable of modeling complex and nonlinear data, to integrate the evaluation of 18 seminal variables plus season, donor, and percentage of viable oocytes. The 3 best AI models for predicting IVEP achieved accuracy rates of 90.7, 75.3, and 79.6%, respectively [47]. The authors emphasized the need to validate this approach with a larger sample size and bulls from different breeds and populations to determine whether these seminal variables can reliably predict embryo yield in diverse datasets.

Surprisingly, the individual effect of the animal, represented by the variable BULL, emerged as the strongest predictor of blastocyst formation, embryo development, and cleavage. When evaluated independently, this variable alone yielded a determination coefficient of up to 0.5218. This raises an important question: What underlies the robust predictive ability of the variable BULL for in vitro performance?

Given that in vitro fertilization involves subjecting sperm to a discontinuous Percoll gradient—removing seminal plasma and semen extender while selecting specific sperm subpopulations—it is conceivable that this individual effect of the animal is carried by these selected sperm. The variable “BULL” in your predictive models’ functions as a categorical identifier that captures the individual effect of each bull, encompassing intrinsic and extrinsic factors that were not directly measured. These factors may include membrane and nuclear proteins [48], microRNAs [49], metabolites [43], and genomic and epigenomic markers [50,51,52], all of which have been linked to bull fertility. Also, factors like cryopreservation protocol, extender, straw concentration, and collection and processing center, among others. Because these relevant bull-level causes were not explicitly accounted for in the model, “BULL” acts as a proxy variable absorbing that unmeasured variability and thus showing a strong predictive power in your models. Our comprehensive dataset, which included 26 sperm variables, likely does not fully encompass the key sperm traits and environmental factors determining in vitro embryo production (IVEP) outcomes. These unmeasured variables collectively contribute to the strong predictive power of the “BULL” variable observed in our models. This finding underscores the necessity for further research aimed at elucidating these underlying intrinsic and extrinsic factors to better understand and predict individual bull fertility variability Two crucial considerations can be made: First, all samples employed in our study were sourced from commercially available semen straws produced by reputable centers, which adhere to standardized quality controls, leading to the discarding of batches below motility and morphology thresholds. Second, all the samples underwent a gradient selection of motile cells before in vitro fertilization and analysis. Additionally, all in vitro production steps were executed by the same technician.

Predictive modeling for cleavage rate produced lower determination coefficients than blastocyst rate predictions. By comparing determination coefficients of predictive models for blastocyst formation, embryo development, and cleavage, we inferred that both the variable BULL and sperm traits have a more substantial role in predicting blastocyst and embryo development rates than in predicting cleavage rates.

As an alternative to our primary hypothesis, we propose that the predictive power of the variable BULL is linked to the sperm traits analyzed, but in a more complex manner—specifically through their interactions. The concept of variable interactions suggests that a particular sperm trait may influence relationships between other traits, ultimately affecting in vitro performance. Understanding this intricate network of relationships between sperm trait levels and IVEP outcomes may require more sophisticated data analysis techniques, e.g., neural networks. The linear model used may offer limited analyses and was used for interpretability. In our previous study [18], we demonstrated the strategic advantage of isolating the effects of individual sperm traits. In that study, motility, acrosome integrity, and mitochondrial membrane potential—traits typically associated only with fertilization—exhibited a delayed effect, influencing embryo development and blastocyst rates without a discernible impact on cleavage rates.

The predictive model for embryo cell number did not reveal an effect associated with the variable BULL. In a recent study conducted by our research team [53], embryos derived from bulls with high in vitro fertility exhibited no significant differences compared to those produced from bulls with low in vitro fertility in terms of morphokinetic parameters, such as cleavage and developmental kinetics. However, notable variations were observed in their molecular profiles, especially at the 8-cell stage. These findings suggested that the individual influence of the animal and sperm traits on blastocyst morphological attributes is either minimal or negligible.

Since the bull’s influence was stronger than any single sperm variable, we developed an explanatory model for the same bull across semen batches. Based on the results, we inferred that individualized models based on sperm traits could serve as a valuable tool for IVEP laboratories, aiding in selecting the best semen batch from a given bull to optimize blastocyst rates. However, it is important to acknowledge that the dataset for individual bull models was smaller (n = 7 or 15 IVEP procedures, depending on the number of batches) compared to the dataset including all bulls (n = 184 IVEP procedures). Although the use of each procedure as an independent data point could potentially inflate significance and R^2^ values due to repeated predictor values across replicate procedures from the same batch, the results from the individual bull models argue against this inflation. We hypothesize that the smaller sample size may contribute to the higher determination coefficients observed in individual bull models relative to those derived from the complete dataset. Importantly, individual models were primarily based on motility variables. This finding aligned with a study on Senepol bulls [46], where motility subpopulations were the only sperm variables incorporated into a predictive IVEP model.

Furthermore, the sperm trait variables used in predictive models varied among individual bulls. This variability has been reported between breeds [54] and between dairy and beef bulls [55]. To mitigate some of this variation, we restricted our analysis to a single breed—the Nellore—given its prominence in Brazil and the IVEP industry. Nonetheless, to the best of our knowledge, our study is the first to reveal residual variability among bulls of the same breed, suggesting that specific sperm traits have varying degrees of relevance for in vitro performance in each bull.

Another intriguing observation was that the relationship between the same sperm trait and blastocyst rate varied among bulls. For example, sperm kinematic subpopulation 4 (SUB 4) exhibited a positive correlation with blastocyst rate for Bull 10 but a negative correlation for Bull 12. This variation contributed to the lower determination coefficients when data from multiple bulls were analyzed collectively, as positive and negative relationships counterbalanced each other. Based on such divergence in sperm trait behavior, we inferred that traits traditionally considered quality indicators or positively correlated with in vivo fertility may have adverse effects under in vitro conditions. A similar finding was central to our previous study [18], which demonstrated that excessively high levels of certain sperm traits could negatively impact in vitro outcomes. In the present study, the bull factor had a comparable role. These findings collectively suggested that sperm traits do not exhibit a simple linear influence on in vitro fertility and that traits conventionally associated with fertility may, under certain conditions, yield detrimental effects in an in vitro environment.

The influence of sperm quantity per straw was investigated in the context of fertility prediction, revealing bull-dependent effects [56,57]. As proposed by Utt [33], this phenomenon aligned with the concept of compensable and non-compensable traits outlined by Saacke [58]. In this context, increasing the number of sperm used in artificial insemination eliminates the correlation between sperm traits and fertility, suggesting that the effect of sperm traits is overshadowed. In IVF, sperm count is standardized based on motile cells; however, other sperm trait levels vary among samples and bulls. This discrepancy may explain variation among bulls in predictive model-contributing variables.

Additionally, it is important to consider that, under in vitro conditions, there is no mechanism to optimize sperm concentration during fertilization, as occurs in vivo [35]. This lack of regulation may explain why certain sperm traits that have negligible effects in the field can have a negative impact in vitro, depending on the bull. Importantly, the predictive models developed in this study, which employ general linear models and regression, accounted only for linear relationships between dependent and independent variables. When predictive models are constructed using linear models and regression, ensuring a minimum level of correlation among variables is imperative [33]. However, our previous work [18] demonstrated that all sperm traits examined exhibit varying degrees of correlation. This may explain why statistical software selects only 1 variable from each analysis for predictive models, as adding correlated variables does not enhance predictive power and may introduce multicollinearity.

To address large sets of correlated and multicollinear variables, e.g., those derived from CASA, principal component analysis (PCA) is a valuable tool [33]. We employed PCA to define sperm subpopulations, and these variables emerged as the strongest predictors in certain individual models, further validating the effectiveness of this analytical approach.

Despite the limitations of our model, the consistent identification of the BULL variable as the dominant factor across different model structures suggests the presence of a strong biological signal. However, the magnitude and statistical properties of this effect may depend on the modeling framework and should therefore be interpreted with caution.

## 5. Conclusions

In conclusion, despite comprehensive sperm analyses, including cytometry and motility assessments, the predictive models explained only a small portion of the variation in embryo production rates. Overall, sperm traits showed limited utility in predicting in vitro embryo production (IVEP), with the bull being the most influential factor. Moreover, the relationship between sperm traits and IVEP success was not consistent across bulls as both positive and negative associations were observed, indicating complex and potentially nonlinear interactions. These findings highlight important limitations of current predictive models based solely on sperm analysis and emphasize the need for more sophisticated approaches that account for individual bull variability. The results challenge the assumption that specific sperm traits universally predict fertility outcomes and suggest that the interactions among these traits are more intricate than previously recognized. This complexity likely reflects inherent differences in sperm characteristics among nulls and their variable influence on IVEP outcomes.

## Figures and Tables

**Table 1:** Dataset variables.

Categorical Variable	Bull
Continuous Variable	Sperm subpopulation: SUB 1, SUB 2, SUB 3 SUB 4
	CASA parameters: VAP, VSL, VCL, ALH, BCF, STR, LIN, TM, WOB, PM, RAPID, MEDIUM, SLOW, and STATIC
	Flow cytometry: Acrosome integrity (AI), plasma membrane integrity (PI), high mitochondrial membrane potential (HMMP), and chromatin damage (CD)
	Sperm preparation for IVF: Motility before selection (MB), motility after selection (MA), sperm concentration (CONC), final volume (FV), and added volume (AV)
Dependent Variable	Cleavage rate, blastocyst rate, embryo development rate, and number of blastomeres

**Table 2:** Descriptions of sperm movement characteristics and spermatozoa kinetic parameters for determining subpopulations.

Subpopulation	Features of Sperm Movements	Characteristics of Sperm Kinetics Parameters
1—Fast and progressive	Fast, progressive, linear sperm with high oscillation	- VCL, VSL, VAP, LIN, STR, ALH, WOB e DANCE with high value.
2—Hiperactivated	Hyperactivated sperm—fast, sinuous with low linearit	- VCL, VSL, VAP, ALH e DANCE with high values. - LIN with low values.
3—Slow and winding	Slow sperm with low progressiveness and linearity and high oscillation	- VAP e VCL with lower values. - LIN e STR with low intermediate values - BCF with high values.
4—Slow and progressive	Slow sperm, however, progressive, and linear, with a low sinuosity trajectory	- VAP, VCL, DANCE, ALH with low values. - STR, LIN e WOB with high values

VAP—Travel speed; VSL—Progressive speed; VCL—Curvilinear speed; ALH—amplitude of lateral head displacement; BCF—flagellum beating frequency; STR—Straightness; LIN—Linearity; WOB—Wobble = (VAP/VCL) × 100; Dance = VCL × ALH.

**Table 3:** Predictive models with selected variables and r^2^adj for blastocyst rate. Only models with significance are shown.

IVP Predictive Model	Methodology	Variables Selected	r^2^_adj_
Blastocyst rate	Complete model	BULL; MA; MEDIUM; STATIC and CLEAVAGE	0.6358
	Equation: −0.7167(Intercept) − 13.9148(Bull 11) − 10.3562x(Bull 8) − 25.687x(Bull 19) − 21.9089x(Bull 13) − 19.9369x(Bull 10) − 12.3049x(Bull 5) − 28.4102x(Bull 21) − 11.5318x(Bull 3) − 25.8215x(Bull 20) − 19.5542x(Bull 16) − 17.9109x(Bull 12) − 12.7077x(Bull 6) − 22.6632x(Bull 18) − 27.0016x(Bull 22) − 9.4295x(Bull 4) − 3.9255x(Bull 2) − 19.4698x(Bull 15) − 26.7895x(Bull 17) − 19.6952x(Bull 7) − 18.7539x(Bull 14) − 32.7695x(Bull 23) − 16.7354x(Bull 9) + 0.1273x(MA) − 0.2055x(MEDIUM) + 0.0621x(STATIC) + 0.452x(CLEAVAGE)		
	Simplified model—removed Bull	SUB1; SUB2; MB; STR; MEDIUM; SLOW and CLEAVAGE	0.4321
	Equation: − 69.9345x(Intercept) + 0.1657x(SUB1) + 0.1256x(SUB2) + 0.1199x(MB) + 0.2055x(STR) − 0.3181x(MEDIUM) + 0.2055x(SLOW) + 0.8198x(CLEAVAGE)		
	Only BULL	Only Bull 1 was eliminated	0.5218
	Equation: 42.881x(Intercept) − 18.831x(Bull 11) − 15.609x(Bull 8) − 29.549x(Bull 19) − 21.651x(Bull 13) − 18.357x(Bull 10) − 11.392x(Bull 5) − 31.149x(Bull 21) − 7.943x(Bull 3) − 29.799x(Bull 20) − 24.502x(Bull 16) − 20.247x(Bull 12) − 14.046x(Bull 6) − 28.843x(Bull 18) − 32.532x(Bull 22) − 9.431x(Bull 4) − 2.537x(Bull 2) − 23.194x(Bull 15) − 28.619x(Bull 17) − 14.737x(Bull 7) − 21.996x(Bull 14) − 39.677x(Bull 23) − 16.731x(Bull 9)		
Embryonic development rate	Complete model	BULL; MA; MEDIUM and STATIC	0.52
	Equation: + 43.771x(Intercept) − 18.291x(Bull 11) − 13.314x(Bull 8) − 35.583x(Bull 19) − 28.838x(Bull 13) − 24.814x(Bull 10) − 15.095x(Bull 5) − 39.407x(Bull 21) − 13.992x(Bull 3) − 37.137x(Bull 20) − 26.075x(Bull 16) − 24.057x(Bull 12) − 15.495x(Bull 6) − 32.662x(Bull 18) − 38.464x(Bull 6) − 11.752x(Bull 4) − 4.496x(Bull 2) − 26.362x(Bull 15) − 36.759x(Bull 17) − 22.985x(Bull 7) − 25.434x(Bull 14) − 49.619x(Bull 23) − 21.438x(Bull 9) + 0.159x(MA) − 0.274x(MEDIUM) + 0.086x(STATIC)		
	Simplified model—removed Bull	SUB 1; AI; MB and MEDIUM	0.05
	Equation: + 34.996x(Intercept) + 0.26x(SUB 1) − 0.249x(AI) + 0.197x(MB) − 0.325x(MEDIUM)		
	Only BULL	Only Bull 1 was eliminated	0.48
	Equation: +53.986x(Intercept) − 18.255x(Bull 11) − 14.855x(Bull 8) − 32.411x(Bull 19) − 25.021x(Bull 13) − 22.442x(Bull 10) − 14.947x(Bull 5) − 37.055x(Bull 21) − 11.794x(Bull 3) − 33.68x(Bull 20) − 28.616x(Bull 16) − 23.804x(Bull 12) − 14.149x(Bull 6) − 32.447x(Bull 18) − 38.091x(Bull 22) − 9.409x(Bull 4) − 2.801x(Bull 2) − 27.018x(Bull 15) − 33.967x(Bull 17) − 17.758x(Bull 7) − 23.825x(Bull 14) − 48.515x(Bull 23) − 18.753x(Bull 9)		
Cleavage rate	Complete model	BULL; SUB 1; SUB 2 and CONC	0.3576
	Equation:77.8038x(Intercept) − 4.4417x(Bull 11) − 1.6185x(Bull 8) − 9.548x(Bull 19) − 2.6755x(Bull 13) +10.7324x(Bull 10) +7.0448x(Bull 5) − 6.9009x(Bull 21) + 12.6803x(Bull 3) − 15.5831x(Bull 20) − 4.1607x(Bull 16) − 0.5951x(Bull 12) + 0.7831x(Bull 6) − 6.1188x(Bull 18) − 4.1293x(Bull 22) − 0.4942x(Bull 4) + 7.4804x(Bull 2) − 0.6405x(Bull 15) − 0.6502x(Bull 17) + 12.4216x(Bull 7) − 4.2284x(Bull 14) − 9.7463x(Bull 23) +0.2026x(Bull 9) + 0x(Bull 1) +0.1624x(SUB 1) − 0.225x(SUB 2) − 0.0981x(CONC)		
	Simplified model—removed Bull	AI and VAP	0.0230
	Equation: 93.967x(Intercept) − 0.179x(AI) − 0.06x(VAP)		
	Only BULL	Only Bull 1 was eliminated	0.2775
	Equation: 78.721x(Intercept) − 11.013x(Bull 11) − 9.024x(Bull 8) − 13.741x(Bull 19) − 5.377x(Bull 13) − 1.261x(Bull 10) + 1.725x(Bull 5) − 10.337x(Bull 21) + 4.182x(Bull 3) − 14.761x(Bull 20) − 7.321x(Bull 16) − 5.633x(Bull 12) − 5.695x(Bull 6) − 14.04x(Bull 18) − 12.969x(Bull 22) − 3.918x(Bull 4) + 0.016x(Bull 2) − 7.19x(Bull 15) − 8.639x(Bull 17) + 1.35x(Bull 7) − 10.159x(Bull 14) − 17.37x(Bull 23) − 4.782x(Bull 9)		
Cell number count	Complete model	MA; MEDIUM and SLOW	0.1283
	Equation: 173.8248x(Intercept) − 0.5752x(MA) + 1.7325x(MEDIUM) + 1.679x(SLOW)		
	Simplified model—removed Bull	MA; MEDIUM and SLOW	0.1283
	Equation: 173.8248x(Intercept) − 0.5752x(MA) + 1.7325x(MEDIUM) + 1.679x(SLOW)		
	Only BULL	*No significant*	-
	*Equation: -*		

Legend: AI = acrosome integrity; MA = motility after selection; MB = motility before selection; SUB1 = subpopulation 1; SUB2 = subpopulation 2; VAP = average path velocity; STR = straightness.

**Table 4:** Predictive model for blastocyst rates from individual bulls and selected variables. Only models with significance are shown.

Bull ID	Methodology	Variables Selected	Filtering Data Base	Blastocyst Rate r^2^_adj_ (n = 184)
Bull 5	Best Model	SUB 1 and VAP	15	0.58
Equation: −61.42x(Intercept) + 2.52x(SUB 1) + 0.25x(MA)				
Bull 21	Best Model	SUB 3; MA; VCL and SLOW	13	0.86
Equation: −31.98x(Intercept) + 0.26x(SUB 3) − 0.23x(MA) − 0.07x(VCL) − 0.12x(SLOW)				
Bull 18	Best Model	SUB 4; CD; MA and CONC	13	0.83
Equation: 12.40x(Intercept) + 0.398x(SUB 4) + 5.64x(CD) − 0.26x(MA) +0.23x(CONC)				
Bull 22	Best Model	SUB 2; AI; CONC and STATIC	9	0.96
Equation: −21.898x(Intercept) − 0.998x(SUB 2) + 0.858x(AI) − 0.36x(CONC) + 0.042x(STATIC)				
Bull 16	Best Model	CONC; LIN and MEDIUM	7	0.98
Equation: +92.844x(Intercept) − 0.772x(CONC) − 1.65x(LIN) + 0.766x(MEDIUM)				
Bull 15	Best Model	SUB 3 and CLEAVAGE	9	0.59
Equation: −76.936x(Intercept) + 1.07x(SUB 3) + 0.908x(CLEAVAGE)				
Bull 14	Best Model	PI; HMMP; MB; CONC; STR; RAPID and MEDIUM	9	0.99
Equation: 245.793x(Intercept) − 0.41x(PI) 0.203x(HMMP) + 0.097x(MB) − 0.875x(CONC) − 2.329x(STR) + 0.345x(RAPID) + 0.077x(MEDIUM)				
Bull 12	Best Model	SUB 4; AI; CD; BCF and CLEAVAGE	10	0.92
Equation: 355.794x(Intercept) − 0.984x(SUB 4) − 4.07x(AI) − 11.75x(CR) + 0.657x(BCF) + 0.268x(CLEAVAGE)				
Bull 17	Best Model	MA; FV; ALH and CLEAVAGE	8	0.99
Equation: −64.68x(Intercept) − 0.07x(MA) + 0.07x(FV) + 8.10x(ALH) + 0.131x(CLEAVAGE)				
Bull 10	Best Model	SUB 4; MB; MA; RAPID	8	0.96
Equation: −31.66x(Intercept) + 049x(SUB 4) + 0.36x(MB) + 0.48x(MA) − 0.20x(RAPID)				
Bull 4	Best Model	MB; MA; RAPID and CLEAVAGE	10	0.80
*Equation: −57.555x(Intercept) + 0.335x(MB) + 0.197x(MA) + 0.509x(RAPID) + 0.661x(CLEAVAGE)*				

Legend: CD = chromatin denaturation; AI = acrosome integrity; PI = plasma membrane integrity; HMMP = high mitochondrial membrane potential; FV = final volume; CONC = concentration; MA = motility after selection; MB = motility before selection; SUB 1 = subpopulation 1; SUB 2 = subpopulation 2; SUB 3 = subpopulation 3; SUB 4 = subpopulation 4; VAP = average path velocity; VCL = curvilinear velocity; STR = straightness; LIN = linearity; ALH = lateral head displacement; BCF = beat cross frequency.

## Data Availability

The original contributions presented in this study are included in the article/Supplementary Material. Further inquiries can be directed to the corresponding author.

## Supplementary Materials

The following supporting information can be downloaded at: https://www.mdpi.com/article/10.3390/biom16040581/s1, Figure S1: Percentage of blastocyst produced from different batches from the same bull; Figure S2: Determination coefficients obtained by the thresholds and gates configurations for the controls and proportion analyses applied during flow cytometer analysis; Figure S3: Flow Cytometer Classification for each fluorescent probe and selected histograms; Table S1: Blastocyst rate models; Table S2: Embryonic development rate model; Table S3: Cleavage rate model; Table S4: Cell number model.

## Abbreviations

The following abbreviations are used in this manuscript:

IVEP	In vitro Embryo Production
CASA	Computer Assisted Sperm Analysis
COC	Cumulus-Oocyte Complex
TCM	Tissue Culture Medium
FCS	Fetal Calf Serum
FSH	Folicule Stimulation Hormone
hCG	Human Chorionic Gonadotrophin
IVM	In vitro Maturation
IVF	In vitro Fertilization
IVC	In vitro Culture
BSA	Bovine Serum Albumin
KSOM	Potassium Simplex Optimized Medium
MB	Motility Before selection
MA	MotilityAfter selection
CONC	Concentration
AV	Added Volume
FV	Final Volume
AI	Acrosomal Integrity
PI	Plasmatic membrane Integrity
HMMP	High Mitochondrial Membrane Potential
CD	Chromatin Denaturation
HCL	Chloridric acid
FSC	Forward scatter
STR	Straightness
VAP	Path Velocity
TM	Total Motility
PM	Progressive Motility
VCL	Curvilinear velocity
ALH	Lateral Head displacement
BCF	Beat-cross frequency
VSL	Straight line Velocity
PVP	Polivinilpirolidone
PCA	Principal Component Analysis
SUB	Subpopulation
