# Supplementary material for: Individual Effect of Bull Prevails over Sperm Characteristics in Predictive Models
Source: Biomolecules. 2026 Apr 14;16(4):581. doi: 10.3390/biom16040581 (PMC13113200; doi:10.3390/biom16040581)
Supplement: Supplementary file 1 [file biomolecules-16-00581-s001.zip › biomolecules-4144702-supplementary.pdf]

Supplementary Data

Table S1: Blastocyst rate models

Models	Methodology	Variables Selected	Blastocyst rate r^2_{adj} (n=184)
1st	Complete model	BULL; MA; MEDIUM; STATIC and CLEAVAGE	0,6358
Equation: -0.7167(Intercept) -13.9148(Bull 11) -10.3562(Bull 8) -25.687*(Bull 19) -21.9089*(Bull 13) -19.9369*(Bull 10) -12.3049*(Bull 5) -28.4102*(Bull 21) -11.5318*(Bull 3) -25.8215*(Bull 20) -19.5542*(Bull 16) -17.9109*(Bull 12) -12.7077*(Bull 6) -22.6632*(Bull 18) -27.0016*(Bull 22) -9.4295*(Bull 4) -3.9255*(Bull 2) -19.4698*(Bull 15) -26.7895*(Bull 17) -19.6952*(Bull 7) -18.7539*(Bull 14) -32.7695*(Bull 23) -16.7354*(Bull 9) + 0.1273*(MA) -0.2055*(MEDIUM) + 0.0621*(STATIC) + 0.452*(CLEAVAGE)*			
2nd	Simplified model - removed Bull	SUB1; SUB2; MB; STR; MEDIUM; SLOW and CLEAVAGE	0,4321
Equation: -69.9345(Intercept) + 0.1657*(SUB1) + 0.1256*(SUB2) + 0.1199*(MB) + 0.2055*(STR) -0.3181*(MEDIUM) + 0.2055*(SLOW) + 0.8198*(CLEAVAGE)*			
3rd	Flow Cytometry	Only CD	0,0134
Equation: 21.048(Intercept) + 1.410*(CD)*			
4th	CASA	BCF and STR	0,0160
Equation: -3.420(Intercept) -0.2979*(BCF) + 0.4316*(STR)*			
5th	Sub-Populations	Only SUB1	0,0123
Equation: 18.267(Intercept) + 0.1585*(SUB1)*			
6th	Flow Cytometry and CASA	CD and ALH	0,0240
Equation: 23.5666(Intercept) + 1.625*(CD) - 0.350*(ALH)*			
7th	Flow Cytometry and Sub-Populations	SUB1 and CD	0,0289
Equation: 16.402(Intercept) + 0.1773*(SUB1) + 1.485*(CD)*			
8th	CASA and Sub-Populations	SUB1 and STR	0,0202
Equation: - 0.509(Intercept) + 0.142*(SUB1) + 0.230*(STR)*			
9th	Flow Cytometry, CASA and Sub-Populations	SUB1; CD and ALH	0,0377
Equation: 18.980(Intercept) + 0.169*(SUB1) + 1.683*(CD) - 0.329*(ALH)*			
10th	Only BULL	Only Bull 1 was eliminated	0,5218

Equation: 42.881(Intercept) -18.831*(Bull 11) -15.609*(Bull 8) -29.549*(Bull 19) -21.651*(Bull 13) -18.357*(Bull 10) -11.392*(Bull 5) -31.149*(Bull 21) -7.943*(Bull 3) -29.799*(Bull 20) -24.502*(Bull 16) -20.247*(Bull 12) -14.046*(Bull 6) -28.843*(Bull 18) -32.532*(Bull 22) -9.431*(Bull 4) -2.537*(Bull 2) -23.194*(Bull 15) -28.619*(Bull 17) -14.737*(Bull 7) -21.996*(Bull 14) -39.677*(Bull 23) -16.731*(Bull 9)*

Legend: CD = chromatin denaturation; MA = motility after selection; MB = motility before selection; SUB1 = subpopulation 1; SUB2 = subpopulation 2; STR = straightness; ALH = lateral head displacement; BCF = beat cross frequency.

Table S2: Embryonic development rate model

**Models**	**Methodology**	**Variables Selected**	**Embryonic Development Rate r^2_{adj} (n=184)**
1st	Complete model	BULL; MA; MEDIUM and STATIC	0,52
Equation: + 43.771(Intercept) - 18.291*(Bull 11) - 13.314*(Bull 8) - 35.583*(Bull 19) - 28.838*(Bull 13) - 24.814*(Bull 10) - 15.095*(Bull 5) - 39.407*(Bull 21) - 13.992*(Bull 3) - 37.137*(Bull 20) - 26.075*(Bull 16) - 24.057*(Bull 12) - 15.495*(Bull 6) - 32.662*(Bull 18) - 38.464*(Bull 6) - 11.752*(Bull 4) - 4.496*(Bull 2) - 26.362*(Bull 15) - 36.759*(Bull 17) - 22.985*(Bull 7) - 25.434*(Bull 14) - 49.619*(Bull 23) - 21.438*(Bull 9) + 0.159*(MA) - 0.274*(MEDIUM) + 0.086*(STATIC)*			
2nd	Simplified model - removed Bull	SUB 1; AI; MB and MEDIUM	0.05
Equation: + 34.996(Intercept) + 0.26*(SUB 1) - 0.249*(AI) + 0.197*(MB) - 0.325*(MEDIUM)*			
3rd	Flow Cytometry	only CD	0.01
Equation: + 28.676(Intercept) + 1.43*(CD)*			
4th	CASA	BCF and STR	0.02
Equation: -3.223(Intercept) - 0.379*(BCF) + 0.556*(STR)*			
5th	Sub-Populations	SUB 1 and SUB 2	0.02
Equation: + 20.128(Intercept) + 0.25*(SUB 1) + 0.145*(SUB 2)*			
6th	Flow Cytometry and CASA	BCF; STR and MEDIUM	0.03
Equation: - 0.797(Intercept) - 0.508*(BCF) + 0.598*(STR) - 0.318*(MEDIUM)*			
7th	Flow Cytometry and Sub-Populations	SUB 1 and CD	0.03
Equation: 22.881(Intercept) + 0.221*(SUB 1) + 1.53*(CD)*			
8th	CASA and Sub-Populations	SUB 1; BCF and STR	0.03

Equation: -4.563(Intercept) + 0.172*(SUB 1) - 0.359*(BCF) + 0.511*(STR)*			
9th	Flow Cytometry, CASA and Sub-Populations	SUB 1 and CD	0.03
Equation: + 22.881(Intercept) + 0.221*(SUB 1) + 1.53*(CD)*			
10th	Only BULL	Only Bull 1 was eliminated	0.48
Equation: +53.986(Intercept) - 18.255*(Bull 11) - 14.855*(Bull 8) - 32.411*(Bull 19) - 25.021*(Bull 13) - 22.442*(Bull 10) - 14.947*(Bull 5) - 37.055*(Bull 21) - 11.794*(Bull 3) - 33.68*(Bull 20) - 28.616*(Bull 16) - 23.804*(Bull 12) - 14.149*(Bull 6) - 32.447*(Bull 18) - 38.091*(Bull 22) - 9.409*(Bull 4) - 2.801*(Bull 2) - 27.018*(Bull 15) - 33.967*(Bull 17) - 17.758*(Bull 7) - 23.825*(Bull 14) - 48.515*(Bull 23) - 18.753*(Bull 9)*			

Legend: CD = chromatin denaturation; MA = motility after selection; MB = motility before selection; SUB1 = subpopulation 1; SUB2 = subpopulation 2; STR = straightness; ALH = lateral head displacement; BCF = beat cross frequency.

Table S3: Cleavage rate model

**Models**	**Methodology**	**Variables Selected**	**Cleavage rate r^2_{adj} (n=184)**
1st	Complete model	BULL; SUB 1; SUB 2 and CONC	0.3576
Equation:77.8038(Intercept) -4.4417*(Bull 11) -1.6185*(Bull 8) -9.548*(Bull 19) -2.6755*(Bull 13) +10.7324*(Bull 10) +7.0448*(Bull 5) -6.9009*(Bull 21) + 12.6803*(Bull 3) -15.5831*(Bull 20) -4.1607*(Bull 16) -0.5951*(Bull 12) + 0.7831*(Bull 6) -6.1188*(Bull 18) -4.1293*(Bull 22) -0.4942*(Bull 4) + 7.4804*(Bull 2) -0.6405*(Bull 15) -0.6502*(Bull 17) + 12.4216*(Bull 7) -4.2284*(Bull 14) -9.7463*(Bull 23) +0.2026*(Bull 9) + 0*(Bull 1) +0.1624*(SUB 1) -0.225*(SUB 2) - 0.0981*(CONC)*			
2nd	Simplified model - removed Bull	AI and VAP	0.0230
Equation: 93.967(Intercept) -0.179*(AI) -0.06*(VAP)*			
3rd	Flow Cytometry	Only AI	0.0158
Equation: 88.031(Intercept) -0.179*(AI)*			
4th	CASA	Only VAP	0.0029
Equation: 77.037(Intercept) -0.047*(VAP)*			
5th	Sub-Populations	*No significant*	-
Equation: -			
6th	Flow Cytometry and CASA	AI and VAP	0.0230
Equation: 93.967(Intercept) -0.179*(AI) -0.060*(VAP)*			

7th	Flow Cytometry and Sub-Populations	SUB 2 and AI	0.0190
Equation: 90.674(Intercept) -0.0903*(SUB 2) -0.184*(AI)*			
8th	CASA and Sub-Populations	Only VAP	0.0029
Equation: 77.037(Intercept) -0.047*(VAP)*			
9th	Only BULL	Only Bull 1 was eliminated	0.2775
Equation: 78.721(Intercept) -11.013*(Bull 11) -9.024*(Bull 8) -13.741*(Bull 19) -5.377*(Bull 13) -1.261*(Bull 10) + 1.725*(Bull 5) -10.337*(Bull 21) + 4.182*(Bull 3) -14.761*(Bull 20) -7.321*(Bull 16) -5.633*(Bull 12) -5.695*(Bull 6) -14.04*(Bull 18) -12.969*(Bull 22) -3.918*(Bull 4) + 0.016*(Bull 2) -7.19*(Bull 15) -8.639*(Bull 17) + 1.35*(Bull 7) -10.159*(Bull 14) -17.37*(Bull 23) -4.782*(Bull 9)*			

Legend: CD = chromatin denaturation; AI = acrosome integrity; MA = motility after selection; MB = motility before selection; SUB1 = subpopulation 1; SUB2 = subpopulation 2; VAP = average path velocity; STR = straightness; ALH = lateral head displacement; BCF = beat cross frequency.

Table S4: Cell number model

**Models**	**Methodology**	**Variables Selected**	**Number Cells r^2_{adj} (n=96)**
1st	Complete model	MA; MEDIUM and SLOW	0.1283
Equation: 173.8248(Intercept) - 0.5752*(MA) + 1.7325*(MEDIUM) + 1.679*(SLOW)*			
2nd	Simplified model - removed Bull	MA; MEDIUM and SLOW	0.1283
Equation: 173.8248(Intercept) - 0.5752*(MA) + 1.7325*(MEDIUM) + 1.679*(SLOW)*			
3rd	Flow Cytometry	*No significant*	-
Equation: -			
4th	CASA	ALH and SLOW	0.1064
Equation: 121.317(Intercept) + 3.399*(ALH) + 1.807*(SLOW)*			
5th	Sub-Populations	*No significant*	-
Equation: -			
6th	Flow Cytometry and CASA	VAP and SLOW	0.1003

Equation: 179.243(Intercept) -0.3389*(VAP) + 1.813*(SLOW)*			
7th	Flow Cytometry and Sub-Populations	*No significant*	-
Equation: -			
8th	CASA and Sub-Populations	ALH and SLOW	0.1064
Equation: 121.317(Intercept) + 3.399*(ALH) + 1.807*(SLOW)*			
9th	Only BULL	*No significant*	-
Equation: -			

Figure S1: Percentage of blastocysts produced from different batches from the same bull.

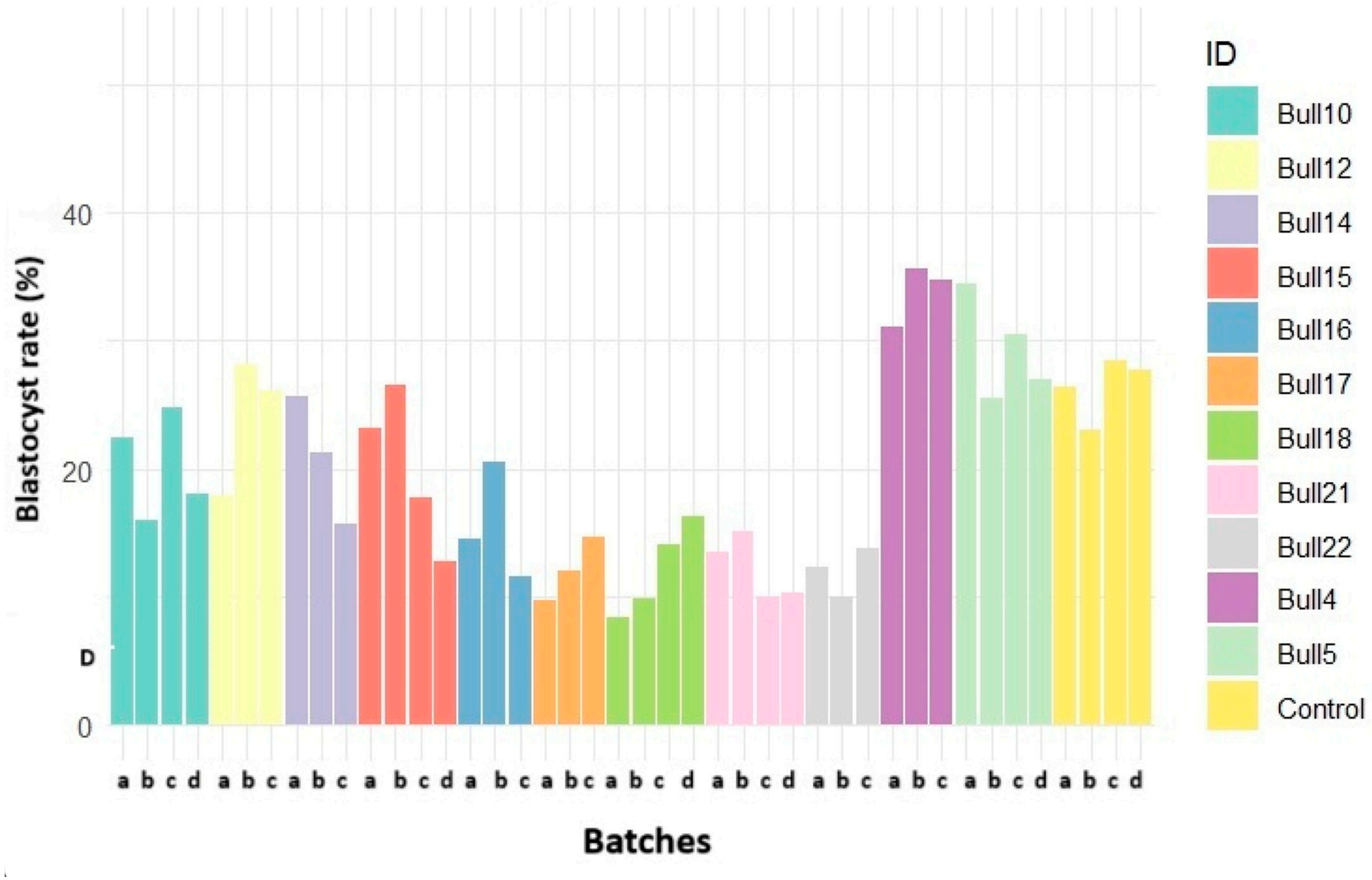

Figure S2: Determination coefficients obtained by the thresholds and gates configurations for the controls and proportion analyses applied during flow cytometer analysis.

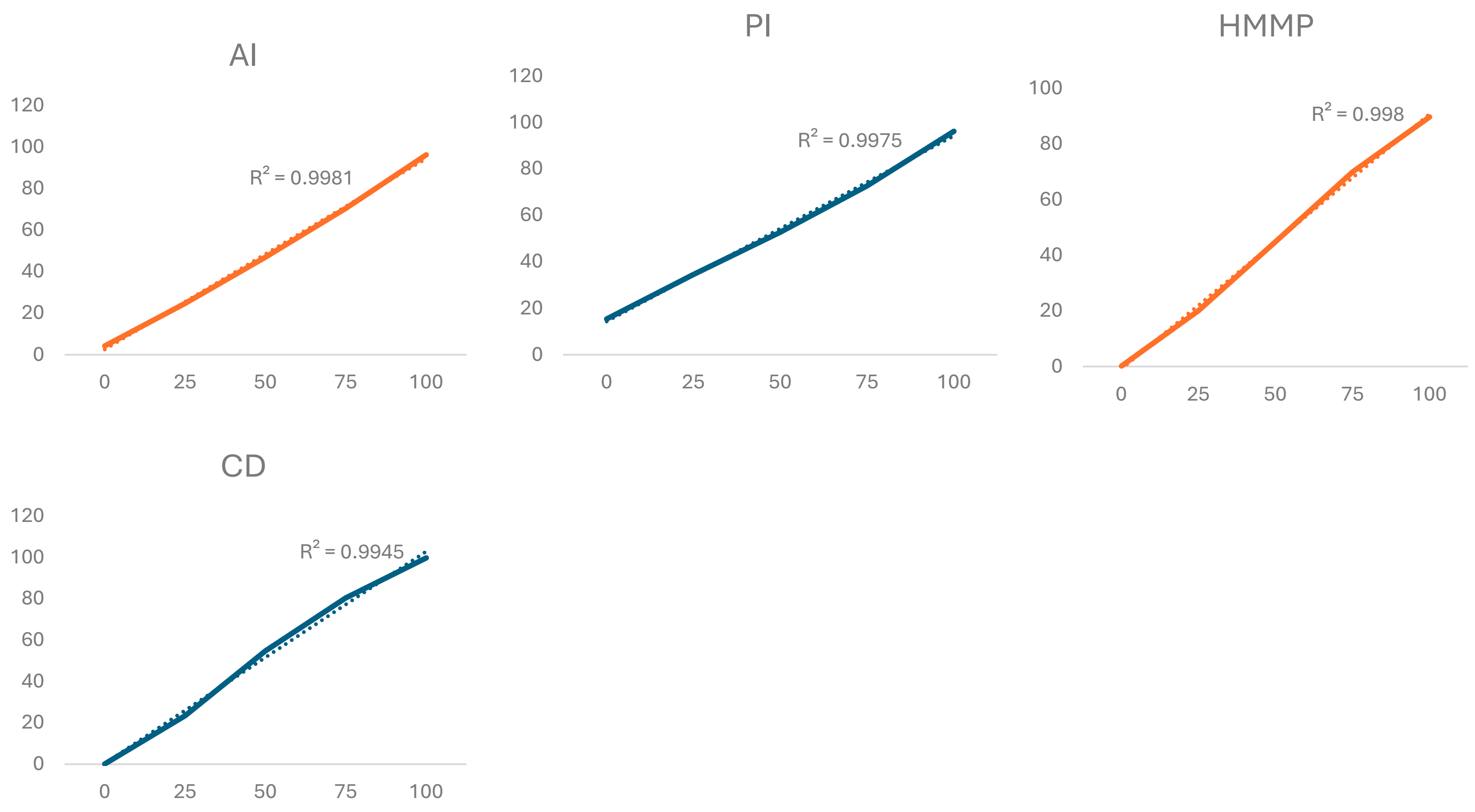

AI – Acrossomal integrity; PI – propidium iodide; HMMP – high mitochondrial membrane potential; CD – chromatin condensation

Figure S3: Flow Cytometer Classification for each fluorescent probe and selected histograms

Total events: Dotplot: Green Fluorescence (log mode) X Foward Scatter<br>Rectangular gate: Selected events					
	Control 1	1:3	1:1	3:1	Control 2
**FITC - PI**	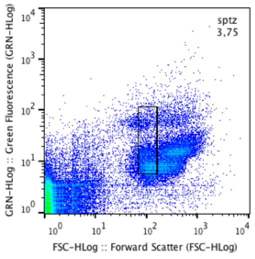	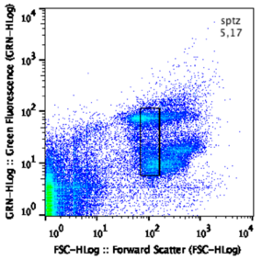	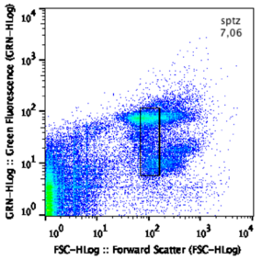	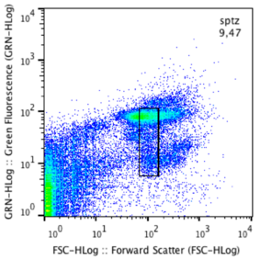	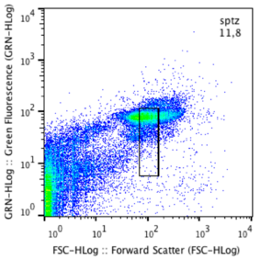
**JC - 1**	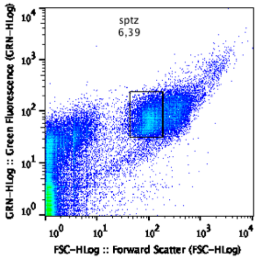	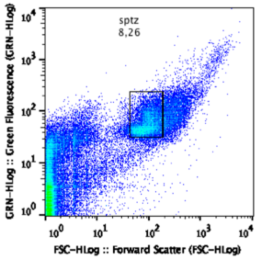	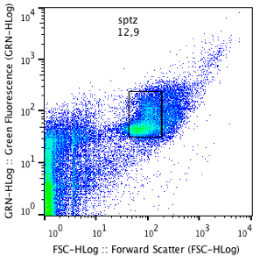	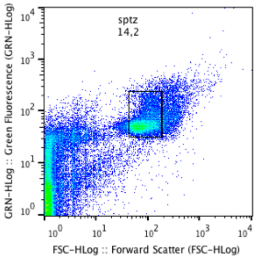	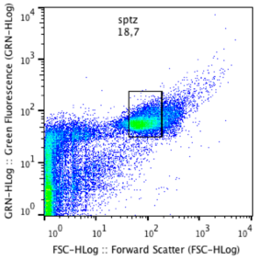
Total events: Dotplot: Red Fluorescence X Foward Scatter<br>Rectangular gate: Selected events					

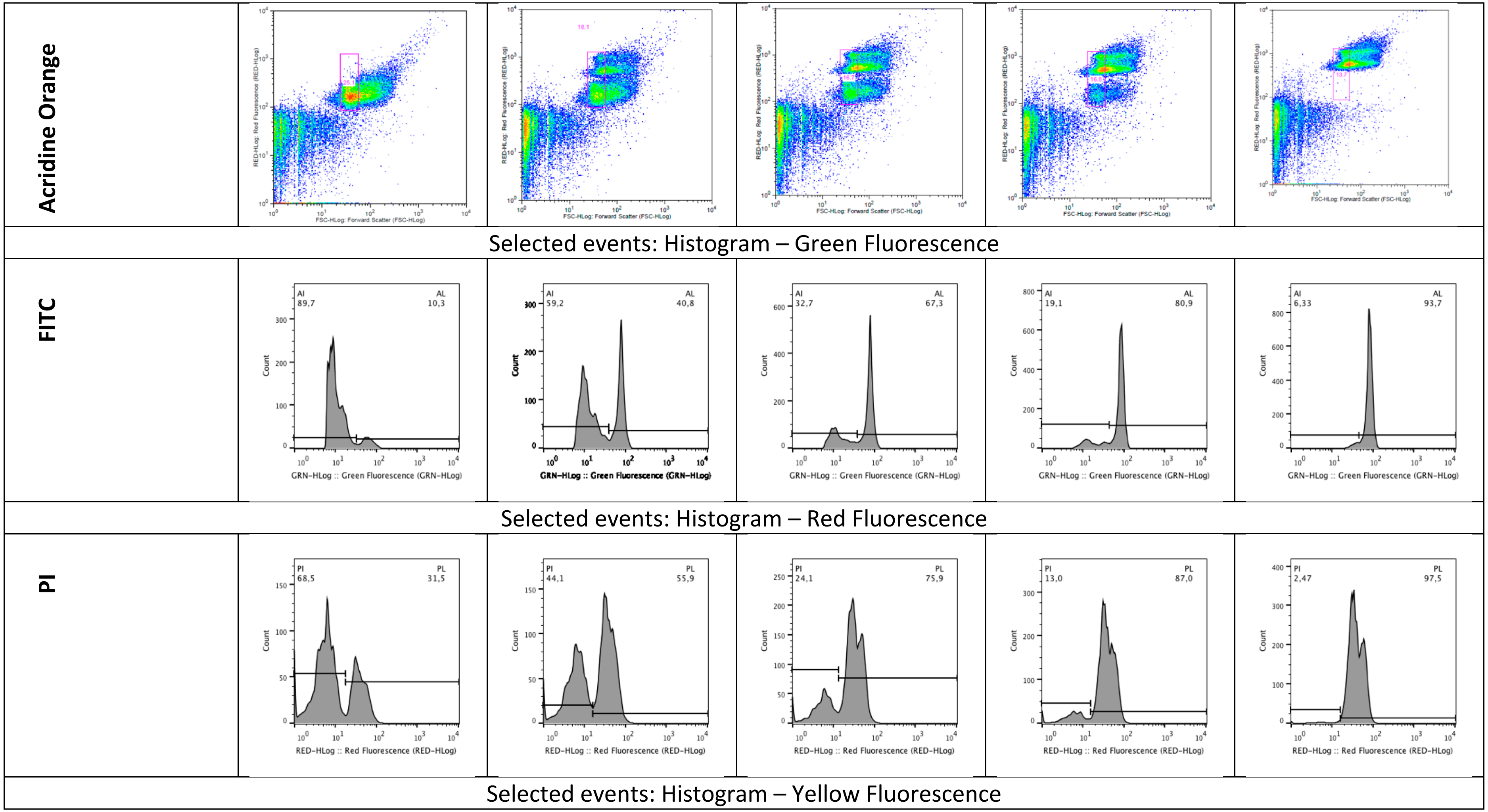
Acridine Orange
FSC-HLog: Forward Scatter (FSC-HLog)
Selected events: Histogram – Green Fluorescence
FITC
AI 89,7 AL 10,3
AI 59,2 AL 40,8
AI 32,7 AL 67,3
AI 19,1 AL 80,9
AI 6,33 AL 93,7
Count
GRN-HLog :: Green Fluorescence (GRN-HLog)
Selected events: Histogram – Red Fluorescence
PI
PI 68,5 PL 31,5
PI 44,1 PL 55,9
PI 24,1 PL 75,9
PI 13,0 PL 87,0
PI 2,47 PL 97,5
Count
RED-HLog :: Red Fluorescence (RED-HLog)
Selected events: Histogram – Yellow Fluorescence

<table>
<tr><td>JC - 1</td>
<td>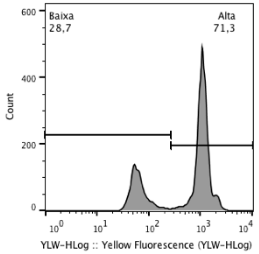
</td>
<td>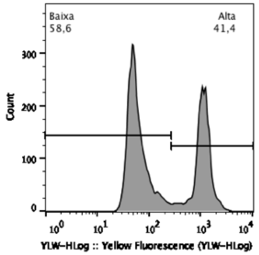
</td>
<td>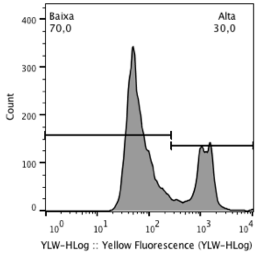
</td>
<td>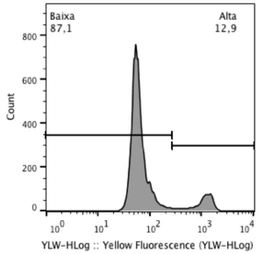
</td>
<td>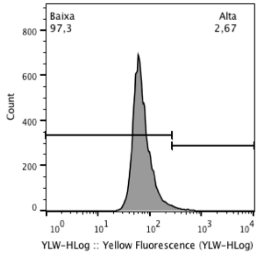
</td></tr>
<tr><td colspan="6">Selected events: Histogram – Alfa TY log</td></tr>
<tr><td>Acridine Orange</td>
<td>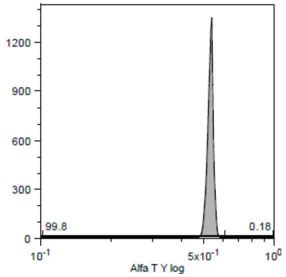
</td>
<td>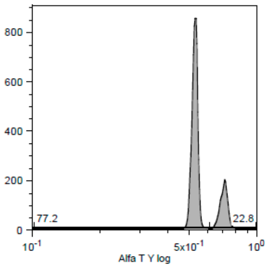
</td>
<td>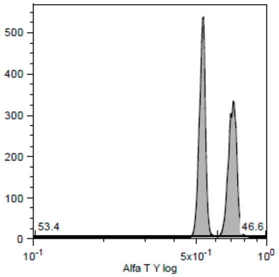
</td>
<td>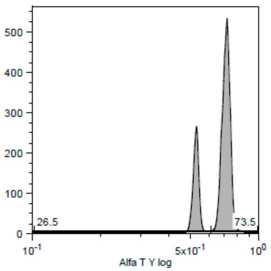
</td>
<td>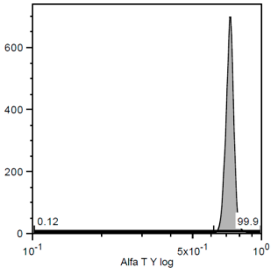
</td></tr>
<tr><td></td><td></td><td></td><td></td><td></td><td></td></tr>
</table>
